# Association between self-compassion and cyber aggression in the COVID-19 context: roles of attribution and public stigma

**DOI:** 10.1186/s40359-023-01100-x

**Published:** 2023-03-10

**Authors:** Qinglu Wu, Tian-Ming Zhang

**Affiliations:** 1 Institute of Advanced Studies in Humanities and Social Sciences, Beijing Normal University, Zhuhai, China; 2 Department of Social Work, Shanghai University, 99 Shangda Road, BaoShan District, Shanghai, 200444 China

**Keywords:** Self-compassion, Attribution of COVID-19, Public stigma of COVID-19, Cyber aggression

## Abstract

Self-compassion is negatively associated with aggressive behaviors. However, the association between self-compassion and cyber aggression toward stigmatized people (e.g., people infected with COVID-19) has not been investigated in the COVID-19 context and the mechanism underlying this association remains underexplored. On the basis of emotion regulation theory and attribution theory, this study examined the indirect effects of self-compassion on cyber aggression toward people infected with COVID-19 through attribution and public stigma of COVID-19. Data were collected from 1162 Chinese college students (415 male, mean age = 21.61 years). Participants completed an online questionnaire including measurement of the key variables and basic demographic information. Results indicated that self-compassion was negatively associated with cyber aggression through the lower attribution of COVID-19 and lower public stigma of COVID-19. A sequential pathway from the attribution of COVID-19 to public stigma of COVID-19 was identified in the relationship between self-compassion and cyber aggression. Our findings are consistent with emotion regulation theory and attribution theory, which posit that emotion regulation strategies are associated with interpersonal mistreatment through cognitive pathways. These findings suggest that emotional self-regulation strategies can be used to reduce cyber aggression toward stigmatized people by reducing attribution and public stigma in the COVID-19 context. Self-compassion improvement could be target for the interventions aiming at alleviating public stigma and interpersonal mistreatment toward stigmatized people.

## Introduction

The outbreak of coronavirus disease 2019 (COVID-19) in 2019 caused great threat to the general public [1]. Individuals with a high risk of COVID-19 exhibited negative attitudes and interpersonal mistreatment (e.g., aggression and stigmatization) in daily life [2, 3], especially demonstrating aggression toward people infected with COVID-19 or accusing them of spreading the disease [4]. Self-compassion, as an effective emotional self-regulation strategy and self-related resource, is helpful in alleviating the negative effects of COVID-19 (e.g., negative emotions, psychological distress, and fear of COVID-19) and aggressive behaviors in daily life [5–8]. Therefore, self-compassion might be associated with aggression toward stigmatized groups, such as individuals infected with COVID-19. To investigate this association and its underlying mechanism, the present study applied emotion regulation theory and attribution theory to examine the indirect effects of self-compassion on cyber aggression through attribution and public stigma of COVID-19.

### Relationship between self-compassion and aggression

Because of the negative effects of the COVID-19 pandemic (e.g., perceived threat, information overload, loneliness, and powerlessness), individuals feel stressed and are more likely to exhibit hostile attitudes and treat others aggressively [2, 9, 10], especially toward stigmatized groups (e.g., individuals infected with or accused of spreading COVID-19) [4, 11]. Cyber aggression has emerged as a common form of aggression due to isolation and decreased social participation caused by the COVID-19 pandemic [3, 4]. Because cyber aggression negatively affected the mental health of both its perpetrators and victims during the COVID-19 pandemic [11, 12], identifying protective factors and psychological resources to reduce its occurrence is necessary.

According to emotion regulation theory [13], self-compassion is an emotional self-regulation strategy, and it may effectively alleviate cyber aggression in the COVID-19 context. Self-compassion is an individuals’ self-related resource and positive response towards themselves when encountering adverse experiences such as inadequacies or difficulties [14, 15]. Moreover, self-compassion is a holistic system including six components that are grouped into compassionate self-responding (self-kindness, common humanity, and mindfulness) and reduced uncompassionate self-responding (decreased self-judgment, isolation, and over-identification) [14, 16]. Instead of responding in an uncompassionate manner, individuals with high levels of self-compassion usually treat themselves with kindness, view adversity as a common shared experience, and maintain a stable mood and a balanced perspective when life difficulties emerge.

The beneficial role of self-compassion in dealing with emotional distress and maintaining positive emotional function has been highly emphasized in studies examining the relationship between self-compassion and aggressive behaviors. A negative association between self-compassion and aggressive behaviors (e.g., verbal and physical aggressive expression) and inclinations has been identified in different populations (e.g., undergraduates and individuals diagnosed with personality disorder) [5, 17, 18]. Self-compassion is helpful in alleviating angry rumination, borderline personality disorder features, and moral disengagement, which subsequently reduce anger and aggressive behavior [5, 18, 19]. As an effective emotional regulation strategy, self-compassion is beneficial for smoothing and regulating negative emotions (e.g., anger) and ruminative thoughts [6, 20]. Therefore, self-compassion can prevent individuals from immersion in negative emotions and prevent their subsequent impulsive and aggressive behaviors toward others [17]. Self-compassionate individuals may be less likely to exhibit hostility and aggressiveness toward people with COVID-19 online.

### Potential indirect effects through attribution and public stigma of COVID-19

Attribution theory provides a perspective for investigating the mechanism underlying the relationship between self-compassion and cyber aggression. Weiner and colleagues (1988) developed a conceptual framework demonstrating that individuals’ attribution of a disease affects their affective responses and behavioral judgment toward groups with the disease. The model posits that people who consider the cause of the disease to be associated with the responsibility and control of those with the disease tend to blame them and endorse increased stigmatization [21]. According to the attribution model of stigma, two common risk factors for aggressive behaviors, namely attribution and public stigma, may play indirect roles in the relationship between self-compassion and cyber aggression toward individuals infected with COVID-19.

Attribution of COVID-19 refers to the cognitive process in which the cause of COVID-19 infection is assigned [22, 23]. Internal attribution, or assigning individual responsibility to those infected with COVID-19, may cause enhanced contempt and aggressive behaviors [24]. A study demonstrated that individuals’ attribution of COVID-19 was linked to their violent behaviors toward those perceived as associated with the disease, such as Asian adults in Western countries [25]. As an adaptive emotional self-regulation strategy and personal resource [15, 26], self-compassion may reduce internal attribution. Studies have identified the negative associations between self-compassion and individuals’ cognitive processes (e.g., rumination and negative cognitive reactions) [20, 27]. Individuals with a high level of self-compassion tend to view suffering as a part of human experience and assume a balanced perspective when facing negative thoughts and emotions instead of isolating themselves and overidentifying during negative experiences. Thus, they are more likely to exhibit positive cognitive reactions and adaptive cognitive processes (e.g., optimism, perspective taking, and positive reframing) and less likely to maintain negative cognitive reactions (e.g., rumination and revenge motivation) [27–29]. Similarly, individuals with a high level of self-compassion may view the negative consequences (e.g., social distancing and quarantine) of the COVID-19 pandemic as an experience shared with others and not be overcome by these adverse experiences. Therefore, they may be less likely to exhibit the causal attribution of COVID-19 (e.g., blame, controllability and responsibility) toward stigmatized people, which further weakens their intentions of aggressive behaviors.

Public stigma of COVID-19 refers to the devaluation of and discriminatory attitudes and beliefs regarding individuals infected with COVID-19 as endorsed by the general population [30]. Social stigmatization toward people associated with COVID-19 increases the likelihood of aggressive behaviors [31]. Because self-compassion may facilitate resilience to stigmatization [32], higher levels of self-compassion might weaken the effect of stigma on aggressive behaviors. Individuals with high levels of self-compassion are less likely to perceive public stigma [33] and more likely to exhibit a prosocial attitude toward others [15, 34]. Individuals with high levels of self-compassion are more likely to demonstrate compassion toward others, which weakens their negative outgroup attitudes [35]. In the context of COVID-19, all Chinese individuals are faced with the negative consequences of the COVID-19 pandemic (e.g., perceived threat and vulnerability to disease, lockdown, social distancing, and quarantine). Thus, when individuals in the COVID-19 context treat themselves kindly, recognize that their experiences are shared by many, and maintain a peaceful mood and balanced perspective of the situation, they are less likely to exhibit hostile or stigmatizing attitudes toward people with COVID-19, which may cause decreased aggressive behaviors.

### Relationship between attribution and public stigma of COVID-19

Studies have provided empirical evidence related to the attribution model in stigma attached to mental illness and infectious disease [36, 37], demonstrating that attribution factors are associated with public stigma. Attribution of the causes of mental illness, such as personal responsibility, predicts negative affective responses and discrimination [38]. The more the individuals perceive those with a disease to be responsible for having that disease, the greater the public stigma is attributed [39]. Several studies have indicated that the more the general population believes that individuals’ infection with COVID-19 is their own responsibility, the more stigmatization they endorse [40, 41]. Negative affect arousal (e.g., fear of contact with COVID-19 or irritation toward individuals infected with COVID-19) is positively associated with elevated levels of stigma [22, 42]. Thus, the association between the attribution and public stigma of COVID-19 was considered in the self-compassion—cyber aggression relationship in this study.

### The present study

Although several studies have addressed aggression during the COVID-19 pandemic, research on the prevention and reduction of aggression has been largely neglected. Identifying psychological factors and intrapersonal resources that may alleviate aggressive behaviors, especially aggression toward individuals infected with COVID-19, is crucial. In addition, delivering interventions on the basis of these factors may help to prevent future mistreatment and stigmatization. According to emotion regulation theory, self-compassion is an effective emotional self-regulation strategy and self-related resource for reducing aggressive behaviors. Moreover, the attribution model of stigma provides a perspective for further examination of the underlying mechanism and potential indirect roles of attribution and public stigma of COVID-19 in the association between self-compassion and cyber aggression. During the COVID-19 pandemic, college students became more reliant on online learning because of lockdown and social distancing policies, which might increase the likelihood of cyberbullying [43, 44]. Thus, in this study, we examined the mechanism underlying the association between self-compassion and cyber aggression toward people infected with COVID-19 among college students. The hypothesized model included three potential indirect effects: self-compassion → attribution of COVID-19 → cyber aggression,self-compassion → public stigma of COVID-19 → cyber aggression; and self-compassion → attribution of COVID-19 → public stigma of COVID-19 → cyber aggression.

## Methods

### Participants and procedure

Data were sourced from a project conducted to investigate the effect of the pandemic fatigue and public stigma of COVID-19 on psychosocial adjustment. Chinese undergraduates and postgraduates aged ≥ 18 years at Beijing Normal University were recruited to participate in the survey. Participants were informed of the research objectives, procedures, and confidentiality policy. Participation was voluntary, and participants were informed that they had the right to withdraw from the survey without any penalty. Participants provided informed consent before responding the online questionnaire. Ethical approval was provided by the research ethics committee of the School of Social Development and Public Policy at Beijing Normal University. Participants who provided a valid response received a small monetary reward (30 RMB, approximately US$ 4.5).

In total, 1317 students provided valid survey responses. After removing surveys with duplicate responses (*n* = 37) and those that failed attention checking (*n* = 118), we obtained 1162 valid surveys (415 men, 747 women). The average age of the participants was 21.61 (SD = 2.81) years. The percentages of undergraduate and postgraduate participants were 65.2% and 34.8%, respectively. The participants’ household monthly income was 19,811.49 RMB (median = 12,000, approximately US $ 1,769.73). Participant characteristics are listed in Table 1.

**Table 1:** Sample characteristics

Characteristic	N	%
*Sex*		
Male	415	35.7%
Female	747	64.3%
*Educational level*		
= High school diploma (undergraduates)	758	65.2%
= Bachelor’s degree (postgraduates)	404	34.8%
*Household monthly income*		
< 10,000	387	33.3%
10,000–20,000	576	49.6%
> 20,000	199	17.1%
*Physical health*		
Poor and very poor	36	3.1%
Fair	276	23.8%
Good and excellent	850	73.1%
*Whether participants or people they know have been infected with COVID-19*		
Yes	138	11.9%
No	1024	88.1%

## Measures

### Self-compassion

Self-compassion was measured using the 12-item Chinese version of the Self-Compassion Scale-Short Form (SCS-SF) [45, 46]. Acceptable reliability of the SCS-SF has been demonstrated in different Chinese populations (e.g., caregivers, adolescents, and college students) [45, 47, 48]. This scale includes six subscales: self-kindness, common humanity, mindfulness, self-judgment, isolation, and over-identification. An example item was “When I feel inadequate in some way, I try to remind myself that feelings of inadequacy are shared by most people.” Responses were obtained on a 5-point Likert scale ranging from 1 (almost never) to 5 (almost always). Higher mean scores indicated higher levels of self-compassion. The Cronbach’s *α* for the SCS-SF in the present study was 0.80.

### Attribution of COVID-19

Attribution of COVID-19 was assessed using a three-item scale developed by our research team. Three scale items ﻿measuring blame, controllability, and responsibility were extracted from the Chinese version of the scale of public stigma toward various infectious diseases [49]. Each item was rated on a 7-point Likert scale ﻿(1 = not at all, 7 = very much), ﻿with higher mean scores indicating agreement with the three internal attributing factors. For example, the item “People with COVID-19 are responsible for their own infection” was used to measure responsibility. The Cronbach’s *α* for this scale in the present study was 0.76.

### Public stigma of COVID-19

Public stigma of COVID-19 was measured using an 11-item scale modified from the Chinese version of an existing scale assessing public stigma of mental illness [37]. The original scale contained two subscales, and we extracted the public stigma dimension with 12 items. Because people infected with COVID-19 were forcibly quarantined in China during the period of data collection, one item, “People with COVID-19 should be quarantined.” was removed [41]. The final scale included 11 items, such as “﻿I am worried that people with COVID-19 will cause harm to others.” Items were rated on a five-point Likert scale (1 = strongly disagree, 5 = strongly agree), with higher mean scores indicating greater public stigma. The Cronbach’s *α* for this scale in the present study was 0.88.

### Cyber aggression

Cyber aggression toward individuals infected with COVID-19 was measured using three items, which were modified from three original items in Chinese used to measure aggressive online behaviors toward Chinese people because of COVID-19 [4]. Responses were rated on a 7-point Likert scale ranging from 1 (not at all) to 7 (very much). An example item was “I would remind people around me to avoid those people who have been infected with COVID-19 online.” Higher mean scores indicated higher levels of cyber aggression toward individuals with COVID-19. The Cronbach’s *α* for this scale in the present study was 0.74.

### Covariates

Covariates in the present study included demographic variables (i.e., age, sex, educational level, and household monthly income) and variables related to health (i.e., physical health) and COVID-19 infection experience of participants or people they knew. Physical health was assessed using one item (“How is your general physical health?”) that participants rated on a 5-point scale ranging from 1 (very poor) to 5 (excellent). COVID-19 infection experience was assessed using one item that asked participants whether they or people they knew had been infected with COVID-19.

### Data analysis

Initial analyses (i.e., descriptive statistics and bivariate correlation) were conducted using SPSS version 24. The SPSS macro PROCESS (Model 6) [50] was used to investigate the direct and indirect effects of self-compassion on cyber aggression through (a) attribution of COVID-19; (b) public stigma of COVID-19; and (c) the pathway from attribution of COVID-19 to public stigma of COVID-19. Age, sex, educational background, household monthly income, physical health, and whether participants or people they knew had been infected with COVID-19 were controlled when model was examined. A resampling approach of bias-corrected bootstrapping (5000 times) with 95% confidence intervals (CIs) was used to estimate the direct and indirect effects of self-compassion on cyber aggression. The effects were considered significant if their CIs did not include 0.

## Results

The descriptive statistics and bivariate correlations of key variables are displayed in Table 2. Self-compassion was negatively associated with the attribution of COVID-19, public stigma of COVID-19, and cyber aggression. Attribution of COVID-19, public stigma of COVID-19, and cyber aggression were positively associated with each other. Harman’s single-factor test was conducted to test for common method variance [51, 52]. Exploratory factor analysis was performed using the items from key variables to verify whether one general factor accounted for the majority of the covariance among measures. The generated principal component analysis revealed that six factors accounted for 59.20% of the total variance. The first unrotated factor explained only 11.55% of the variance in data, suggesting that the common method bias did not substantially affect the model.

**Table 2:** Descriptive statistics and correlations among key variables

	Self-compassion	Attribution of COVID-19	Public stigma of COVID-19	Cyber aggression
Self-compassion	–			
Attribution of COVID-19	− .08^**^	–		
Public stigma of COVID-19	− .28^***^	.53^***^	–	
Cyber aggression	− .16^***^	.54^***^	.53^***^	–
Mean	3.35	2.94	2.68	2.36
Standard deviation	0.54	1.38	0.80	1.25
Range	1.5–5	1–7	1–5	1–7

^*^ *p* < .05, ** *p* < .01, *** *p* < .001

**Fig. 1:**
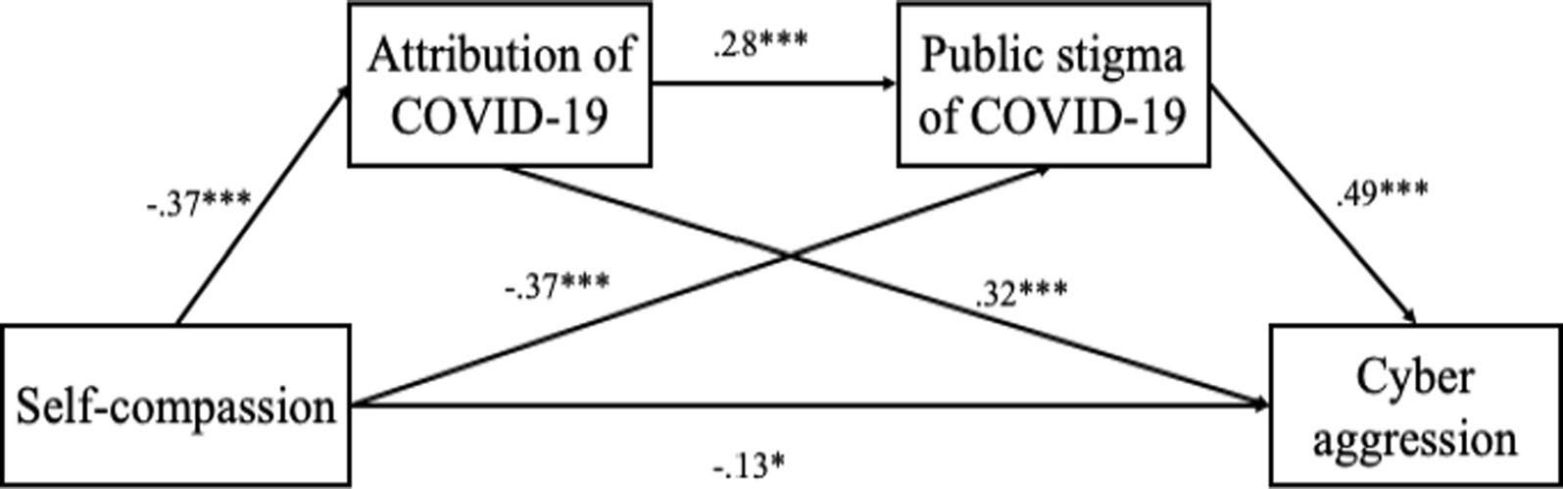
Paths and standardized path coefficients for hypothesized model

The specific direct and indirect effects of self-compassion on cyber aggression are displayed in Table 3. Self-compassion was indirectly associated with cyber aggression through the attribution of COVID-19 (*b* =  − 0.121, *SE* = 0.029, 95% CI =  − 0.180 to − 0.066), public stigma of COVID-19 (*b* =  − 0.180, *SE* = 0.027, 95% CI =  − 0.236 to − 0.129), and attribution and public stigma of COVID-19 in sequence (*b* =  − 0.052, *SE* = 0.013, 95% CI =  − 0.079 to − 0.029]). Details of the specific paths and unstandardized path coefficients are presented in Fig. 1.

**Table 3:** Direct and indirect effects of self-compassion on cyber aggression

	Unstandardized parameter estimate	*S.E*	Bias-corrected CI (95%)	
			Lower	Upper
Direct effect	− .126	.059	− .242	− .010
Indirect effects				
SC → AT → CA	− .121	.029	− .180	− .066
SC → PS → CA	− .180	.027	− .236	− .129
SC → AT → PS → CA	− .052	.013	− .079	− .029

*SC* Self-compassion, *AT* Attribution, *PS* Public stigma, *CA* Cyber aggression

## Discussion

Our findings are in agreement with those of previous studies indicating that self-compassion is beneficial for individuals’ well-being and that of others [15, 53]. Compassionate people alleviate their own emotional difficulties when facing COVID-19-related stress and are less likely to develop internal attribution, stigmatize others, or exhibit aggressive behaviors toward individuals infected with COVID-19. Our findings support emotion regulation theory and attribution theory by demonstrating that self-compassion is an effective emotional self-regulation strategy that associated with reduced cyber aggression toward stigmatized people in the COVID-19 context, and decreased causal attribution and stigma of COVID-19 are critical in this mechanism. Our findings regarding the indirect effect of self-compassion on cyber aggression suggest that self-compassion is useful for modifying individuals’ maladaptation to life stress and challenges caused by the COVID-19 pandemic (e.g., cyber aggression toward stigmatized people).

### Indirect effects of self-compassion on cyber aggression

Indirect effects of self-compassion on cyber aggression are through attribution and public stigma of COVID-19. This finding supports that self-compassion is associated with individuals’ cognitive processes, especially how they view and interpret their negative experiences and their attitudes [20, 27]. Self-compassion was indicated to be negatively linked to attribution of COVID-19 in the present study. Individuals with a high level of self-compassion are less likely to experience negative affect (e.g., irritation, fear, and sadness) or adverse cognitive reaction under challenging circumstances [27, 54]. Thus, if the general population experiencing COVID-19 exhibits greater self-compassion, it is less likely to attribute the control of COVID-19 infection internally by assigning responsibility to those with COVID-19.

Consistently with prior studies, the results of the current study demonstrated that self-compassion was negatively associated with stigma [55–57]. Self-compassionate individuals are kind toward themselves, view individual experiences as part of collective experience, and are less likely to judge the shortcomings of other people [58]. Therefore, self-compassionate individuals may view the inconvenience and emotional distress caused by the COVID-19 pandemic as a common experience (including of the general public and stigmatized individuals) or collective trauma. Moreover, these individuals can maintain a balanced perspective when experiencing negative thoughts and emotions and are less likely to isolate themselves from others or overidentify [20, 58]. Thus, self-compassion might provide psychological resilience to endorsing stigmatizing attitudes [33, 59], and self-compassionate people are less likely to exhibit hostile attitudes and stigmatizing notions toward people infected with COVID-19, which may alleviate cyber aggression toward this stigmatized group.

Self-compassion is associated with cyber aggression through a sequential pathway from the attribution and public stigma of COVID-19, which supports attribution theory [21, 23]. This theory emphasizes that biased responses are decided by a cognitive process: individuals assign casual factors (e.g., controllability, responsibility, and blame) to an individual’s illness that affect their likelihood of exhibiting hostile attitudes and behaviors [60]. Those who exhibit more agreement with attributing factors have higher levels of public stigma and exhibit higher levels of cyber aggression [22, 40, 43]. This relationship is also exhibited by the general population as a whole. Because self-compassion is helpful to alleviates internal attribution [27], it may reduce the negative impact of attribution of COVID-19, which further weakens public stigma and aggressive behaviors.

### Limitations

This study has some limitations. First, because the present study has a cross-sectional design, it could not examine causal relationships among self-compassion, attribution, public stigma, and cyber aggression. Future studies could employ a longitudinal design to examine the mediating roles of attribution and public stigma of COVID-19 in the relationship between self-compassion and aggression. Second, the sample of Chinese college students in the present study limits the generalizability of the findings to other populations. To improve research diversity, the framework of the present study could be applied in other populations (e.g., individuals losing loved ones or encountering severe economic hardship caused by the COVID-19 pandemic) in different developmental stages and cultures. Third, the present study examined only cognition-based mechanisms (attribution and public stigma) between self-compassion and cyber aggression. Self-compassion affects cyber aggression through other cognitive, emotional, or moral pathways (e.g., angry rumination and moral disengagement) [5, 19]. Future research could comprehensively examine pathways linking self-compassion and cyber aggression. Fourth, factors investigating the indirect effect of self-compassion on cyber aggression are not completely identified. To comprehensively investigate the mechanism underlying the relationship between self-compassion and cyber aggression, ecological systems and influential factors at other levels (e.g., parent–child communication and aggression at school) could be considered [61, 62]. Fifth, Internet-related experiences (e.g., problematic Internet use and average time online) related to cyber aggression were not measured in this study [63, 64]. Future research should control for the potential effect of this covariate on cyber aggression.

### Implications

Our findings have theoretical and practical implications. They contribute to the understanding of how self-compassion is associated with interpersonal mistreatment toward stigmatized people in the COVID-19 context. On the basis of emotion regulation theory and the attribution model of public stigma, our study demonstrated the importance of cognitive pathways (attribution and public stigma) when applying emotion regulation strategies for dealing with cyber aggression. These pathways shed light on the cognitive-based mechanism underlying the emotional regulation/dysregulation–interpersonal mistreatment in the aggression (prevention) studies [65, 66]. This research provides insights into the function of self-compassion in stigma (especially public stigma) and aggression reduction and prevention from a perpetrator’s perspective. Studies have verified that self-compassion is helpful in reducing internalized stigma from the perspective of victims [56, 57]. Some researchers argue that self-compassion weakens the effects of public stigma on self-stigma and other negative outcomes [59]. Our findings broaden the application of research on the relationship between self-compassion and stigma, which mainly focuses on the benefits of self-compassion for stigmatized populations [56, 57]. Our study expands previous research that self-compassion is beneficial to perpetrators and can reduce general public stigmatization. Self-compassion promotion is helpful in providing relief and reducing threat perception [67], and individuals’ ability to care for and comfort themselves is beneficial to interpersonal interaction. Our findings also provide practical implications. Because self-compassion is helpful in alleviating negative cognition (e.g., attribution), attitudes (e.g., public stigma), and behaviors (e.g., verbal aggression) related to environmental events (e.g., the COVID-19 pandemic), self-compassion and psychoeducation programs should be used to improve self-compassion and reduce public stigma of COVID-19 [68, 69].

## Data Availability

The datasets generated and/or analysed during the current study are not publicly available due to ethical issues but are available from the corresponding author on reasonable request.
